# Quantifying latent social motivation and its associations with joint attention and language in infants at high and low likelihood for autism spectrum disorder

**DOI:** 10.1111/desc.13336

**Published:** 2022-10-31

**Authors:** Isabella C. Stallworthy, Daniel Berry, Savannah Davis, Jason J. Wolff, Catherine A. Burrows, Meghan R. Swanson, Rebecca L. Grzadzinski, Kelly Botteron, Stephen R. Dager, Annette M. Estes, Robert T. Schultz, Joseph Piven, Jed T. Elison, John R. Pruett, Natasha Marrus, The IBIS Network

**Affiliations:** 1 Institute of Child Development, University of Minnesota, Minneapolis, Minnesota, USA; 2 Department of Psychiatry, Washington University School of Medicine in St. Louis, St Louis, Missouri, USA; 3 Department of Educational Psychology, University of Minnesota, Minneapolis, Minnesota, USA; 4 Department of Pediatrics, University of Minnesota, Minneapolis, Minnesota, USA; 5 School of Behavioral and Brain Sciences, University of Texas at Dallas, Richardson, Texas, USA; 6 Department of Psychiatry, University of North Carolina at Chapel Hill, Chapel Hill, North Carolina, USA; 7 Departments of Radiology and Bioengineering, University of Washington, Seattle, Washington, USA; 8 Department of Speech and Hearing Sciences, University of Washington, Seattle, Washington, USA; 9 Department of Pediatrics, University of Pennsylvania, Philadelphia, Pennsylvania, USA

**Keywords:** autism, infancy, joint attention, language, social motivation

## Abstract

Social motivation—the psychobiological predisposition for social orienting, seeking social contact, and maintaining social interaction—manifests in early infancy and is hypothesized to be foundational for social communication development in typical and atypical populations. However, the lack of infant social-motivation measures has hindered delineation of associations between infant social motivation, other early-arising social abilities such as joint attention, and language outcomes. To investigate how infant social motivation contributes to joint attention and language, this study utilizes a mixed longitudinal sample of 741 infants at high (HL = 515) and low (LL = 226) likelihood for ASD. Using moderated nonlinear factor analysis (MNLFA), we incorporated items from parent-report measures to establish a novel latent factor model of infant social motivation that exhibits measurement invariance by age, sex, and familial ASD likelihood. We then examined developmental associations between 6- and 12-month social motivation, joint attention at 12–15 months, and language at 24 months of age. On average, greater social-motivation growth from 6–12 months was associated with greater initiating joint attention (IJA) and trend-level increases in sophistication of responding to joint attention (RJA). IJA and RJA were both positively associated with 24-month language abilities. There were no additional associations between social motivation and future language in our path model. These findings substantiate a novel, theoretically driven approach to modeling social motivation and suggest a developmental cascade through which social motivation impacts other foundational skills. These findings have implications for the timing and nature of intervention targets to support social communication development in infancy.

## 1 | WHAT IS SOCIAL MOTIVATION?

Human social abilities are thought to be subserved in part by a fundamental motivation to engage with others, especially early in development (Chevallier et al., 2012). Social motivation refers to a collection of psychobiological characteristics that predispose individuals to: (1) preferentially *orient* to the social world, (2) take *pleasure* in social interactions, (3) *seek out* social exchanges, and (4) work to foster and *maintain* social interactions and bonds (Chevallier et al., 2012). According to prominent theoretical accounts (Chevallier et al., 2012; Over, 2016), social motivation manifests behaviorally through socially oriented attention processes and the experience of reward in the context of social interaction. Task-based functional neuroimaging studies support a role for the orbitofrontal-striatum-amygdala reward neurocircuitry in manifestations of social motivation, as demonstrated by neural responses to social rewards in school-aged children and adults (e.g., Dubey et al., 2020; Gordon et al., 2016; Schirmer et al., 2008).

Social motivation is theorized to be present early in infancy, prior to later-emerging aspects of social cognition (Chevallier et al., 2012), and several typical infant social behaviors reflect underlying social motivation. Newborn human infants preferentially orient to the salient social features of their environments, such as the faces, voices, and smells of their caregivers, facilitating provision of care and survival (see Shultz et al., 2018). As infants age, they continue to orient to face-like stimuli (Goren et al., 1975), biological motion (Simion et al., 2008), and human voices (Decasper & Fifer, 1980) compared to nonsocial control stimuli. Within the first few months of life, the repertoire of behaviors allowing expression of social motivation grows, as infants begin to smile socially (Messinger & Fogel, 2007), imitate basic facial expressions (Bjorklund, 1987), participate in reciprocal social exchanges (Beebe et al., 2016), and spend more time attending to socially relevant stimuli (Frank et al., 2012; W. Jones & Klin, 2013).

## 2 | DEVELOPMENTAL CASCADES OF SOCIAL MOTIVATION

Social motivation in infancy may exert developmental cascade effects, reflecting the “cumulative consequences for development of the many interactions and transactions occurring in developing systems that result in spreading effects across levels, among domains at the same level, and across different systems or generations” (Masten & Cicchetti, 2010; p. 491). For instance, high social motivation may increase the likelihood of rich social experiences and their related affordances, stimulating further opportunities for learning in other domains (e.g., language). Social motivation has been hypothesized to either enable or constrain social experiences and learning early in life, across the typical-to-atypical continuum, to influence later outcomes (Chevallier et al., 2012). In the case of autism spectrum disorder (ASD), a highly heritable neurodevelopmental disorder characterized by pervasive challenges with social interaction and communication, decreased social motivation is hypothesized to contribute to these core features (Chevallier et al., 2012). Although the median age of diagnosis in the USA is 38 months of age (Maenner, 2021), the social behavioral signs of ASD likelihood may emerge within the first year of life (e.g., W. Jones & Klin, 2013; M. Miller, Iosif, et al., 2017; Nyström et al., 2019; I. Stallworthy et al., 2021). Past work suggests that early disruptions in social motivation and associated alterations in reward processing may help explain, and in part give rise to, the subsequent social cognitive and social communication deficits characteristic of ASD (Chevallier et al., 2012; Clements et al., 2018; Dawson et al., 2002, 2005; Parish-Morris et al., 2021; Schultz, 2005; Van Etten & Carver, 2015). A deeper understanding of the role of early social motivation in typical and atypical development will therefore further knowledge about the building blocks of social communication. Below, we highlight some existing evidence supporting the notion of a cascade in which early social experiences afforded by social motivation might influence the subsequent development of pivotal social skills, such as joint attention, as well as core adaptive outcomes, such as language.

### 2.1 | Social motivation and joint attention

Joint attention or orienting to objects in the environment with another person, is an important milestone for typical social cognitive development (Mundy & Newell, 2007). Near the end of the first year of life, infants begin reliably following the attention-directing cues of others, such as following gaze and points (Carpenter et al., 1998; Mundy et al., 2007), before using their own cues to direct the orienting of others (Mundy et al., 2007). These two forms of joint attention, responding to joint attention (RJA) and spontaneously initiating joint attention (IJA), may be shaped by abilities and experiences earlier in ontogeny (Mundy et al., 2009; Mundy & Newell, 2007). For instance, RJA and IJA draw on more basic processes of orienting to others’ faces and processing another’s gaze cues (e.g., Brooks & Meltzoff, 2005; Mundy, 2018) and may vary as a function of socioeconomic resources (Reilly et al., 2020).

Tomasello et al. (2005) proposed a developmental framework in which early emerging social motivation contributes to subsequent development of joint attention abilities as part of an adaptive system subserving human social engagement. Within this framework, joint attention (i.e., triadic interaction) develops as a consequence of early dyadic social interactions entailing manifestations of social motivation, such as sharing of emotion and actions during simple routine tasks (Carpenter et al., 1998; Tomasello, 1999). Empirical work supports associations between social motivation and joint attention, with evidence of concurrent correlations between aspects of infant social motivation in dyadic contexts (e.g., smiling and vocalizing) and triadic competencies (e.g., joint looking to an object, following a gaze or point; Striano & Rochat, 1999), as well as associations between positive emotional affect in social interactions and IJA (Vaughan et al., 2003) toward the end of the first year of life. Studies have also found diminished RJA (I. Stallworthy et al., 2021; Sullivan et al., 2007) and IJA (Nyström et al., 2019) in infants with ASD, a disorder theoretically associated with atypical social motivation (Chevallier et al., 2012; Phillips et al., 2019). Additionally, Salley et al. (2016) found that social motivation-related behaviors during interactions (i.e., smiling, vocalizing, and eye contact) at 4 months of age were positively associated with future IJA at 18 (but not at 12) months of age for typically developing infants (Salley et al., 2016).

### 2.2 | Joint attention and language

Developmental associations between joint attention abilities and language outcomes are well-established, supporting the theory that joint attention provides a foundation for the development of symbolic thought as well as for learning and producing words (Baldwin, 1995; Tomasello et al., 2005). Past work studying typical development finds associations between joint attention (including items referenced by mothers during joint attentional focus), infants’ word learning (Baldwin, 1991), future vocabulary (Carpenter et al., 1998), word production (Tomasello & Farrar, 1986), and word comprehension (Carpenter et al., 1998). Studies also find that during typical development both RJA (Brooks & Meltzoff, 2005, 2015; Delgado et al., 2002; Morales et al., 2000; Mundy et al., 2007, Mundy et al., 2003) and IJA (Mundy et al., 2007; Mundy et al., 2003) around the first year are associated with future language abilities in toddlerhood, with meta-analysis showing that RJA may more strongly relate to language than IJA (Bottema-Beutel, 2016). Some evidence suggests that, more specifically, within episodes of joint attention, infant sustained attention (gaze for at least 3 s) to objects may be especially important for predicting later vocabulary (Kannass & Oakes, 2008; Yu et al., 2019). Additionally, other work has found positive associations between joint attention and language in toddlers with ASD (Adamson et al., 2019; Bottema-Beutel, 2016; Loveland & Landry, 1986; Mundy et al., 1990; D. S. Murray et al., 2008). Furthermore, some successful interventions targeting joint attention in young children with ASD result in language improvements (Kasari et al., 2008, 2012) conditional on joint attention gains (Bono et al., 2004).

### 2.3 | Social motivation and language outcomes

In addition to contributing to joint attention, social motivation may enhance language development through several mechanisms. Infants with high social motivation may be predisposed to find the act of communication rewarding (Syal & Finlay, 2011), leading them to pursue social interactions that provide opportunities for language exposure. Prelinguistic social engagement within culturally specific social routines that include back-and-forth exchange (e.g., feeding and interactive games; (Hsu et al., 2014) is thought to form the basis for future functional communication (Bruner, 1974; Su et al., 2020; Tomasello, 1992, 2009). These routines provide a shared referential context for specific aspects of language ability, such as vocal imitation and understanding of symbolic representation, and the pragmatic communicative aspects of language that depend on the social feedback young children receive within these adult-led social settings (Tomasello, 1992). Additionally, these socially contingent interactions provide a rewarding context that enables language learning, as articulated by the social gating hypothesis (Kuhl, 2007, 2011; Kuhl et al., 2014).

Early social motivation may also help infants both seek out and tune in to adults’ attention and speech (Shriberg et al., 2011; Su et al., 2020; Tomasello, 1992), including the highly prosodic, drawn-out speech known as child-directed speech (Fernald, 1985), which facilitates language learning (Nelson et al., 1989). This form of engagement also influences phonetic perceptual narrowing, tailoring infants’ language perception to their native language within the first year of life (Kuhl et al., 2003), and which has been found to shape word learning (Hollich et al., 2000; Shneidman et al., 2009; Tomasello, 2000). Greater social motivation during these early sensitive periods could thus afford experiences enhancing key perceptual development processes important for language learning. Lastly, high levels of infant social motivation could increase adults’ efforts to talk to their children during social interactions. Parents may engage more when their infants display more explicit social attunement (L. Murray & Trevarthen, 1986; Warlaumont et al., 2014), which may then reinforce learning and increase the likelihood of future language-rich social interactions.

In sum, existing work suggests that social motivation is present early in infant social behavior and may exert cascading effects on the development of both joint attention and language. Despite this theorized role for early social motivation, there is relatively little work measuring and investigating social motivation in infancy (Marrus et al., in press), a period of rapid social development when signs of atypical social development relevant for ASD first emerge (e.g., W. Jones & Klin, 2013; M. Miller, Iosif, et al., 2017).

### 2.4 | Measuring social motivation

Despite the theorized role for social motivation in development, there is a lack of assessments explicitly designed to measure social motivation in infancy (except for Marrus et al., in press; Marrus et al., 2017). Past studies have mostly focused on a single aspect of the multi-faceted social-motivation construct, such as social orienting (e.g., E. J. H. Jones et al., 2017; although see Vernetti et al., 2018). However, theory suggests that social motivation encompasses many aspects of engagement with the social world (Chevallier et al., 2012). To date, there has been little work operationalizing social motivation, translating this theory of a unified social-motivation construct into a testable statistical model.

Existing parent-report measures of social motivation have been developed for toddlers and older children (e.g., Cohen et al., 2003; Phillips et al., 2019). Recent work in infants has selected face-valid items for a social-motivation composite index drawn from common parent-report questionnaires (Marrus et al., 2017; Marrus et al., in press). Leveraging this work, we suggest that like many other psychological phenomena, social motivation can be modeled as a latent construct, or one that cannot be directly observed but can be indexed by many different observable indicators, including items from existing developmental assessments. Accordingly, an underlying latent social-motivation factor may give rise to associations between observable indicators (Bollen, 2002), specifically those representing the aspects of social motivation defined by Chevallier et al. (2012). Modeling social motivation as a latent variable is advantageous as it allows us to test empirically a theoretically based construct while accounting for measurement error. Estimation of such a latent factor could then be leveraged to explore the relations between social motivation and developmental outcomes.

Within a heterogeneous population, it is possible that indicators of latent social motivation could exhibit measurement *non*-invariance, meaning they function differently depending on certain participant characteristics (Bauer, 2017). For instance, the utility of some indicators for social motivation (e.g., “reaches arms toward you to be picked up”) could change with age as infants develop new capacities (see Supporting Information for more details). This is one form of measurement bias that, if not accounted for, can limit our abilities to establish a psychometrically sound latent model for investigating developmental relations. Conversely, measurement *in*variance (MI) refers to the ideal situation in which a scale or construct provides the same results across several different samples or populations who have similar levels of a given construct (APA, 2015). Testing and correcting for MI is becoming increasingly common in developmental science to establish validity and comparability of measurements across individuals and groups (Bauer, 2017), especially within heterogeneous samples that differ by age and demographic variables (e.g., DeJoseph et al., 2021; Sifre et al., 2021).

## 3 | THE CURRENT STUDY

In the present study, we tested for potential cascade, or spreading, effects of social motivation measured dimensionally across the continuum of typical to atypical development during the first year of life. The goals of this study were twofold: (1) To establish a latent factor model of social motivation at 6 and 12 months that could be used to analyze developmental relations by first testing and adjusting for any measurement non-invariance as a function of age, sex, and familial ASD likelihood in our indicators of social motivation and (2) To investigate the potential downstream relations between infant social motivation and joint attention at 12–15 months and language abilities at 24 months for infants at high and low likelihood for ASD. This study takes a dimensional approach in a large, heterogeneous sample of infants at high and low familial likelihood of ASD to examine associations between continuously distributed domains of functioning across the spectrum of typical to atypical development (Cicchetti, 1984).

We hypothesized that items spanning all the theorized social-motivation elements (orienting, liking, wanting, seeking, and maintaining) from common parent-report questionnaires would together represent a single latent factor across 6 and 12 months of age. To characterize more accurate developmental relations, we tested and adjusted for measurement non-invariance, which we explored for age, sex, and familial likelihood. We hypothesized cascading effects evidenced by positive longitudinal associations between 6-month social motivation, as well as growth in social motivation from 6 to 12 months, and IJA and RJA, both measured at 12–15 months of age. Additionally, we hypothesized positive relations between 6-month social motivation and growth in social motivation with receptive and expressive language outcomes at 24 months of age. Based on prior work, we also hypothesized positive relations between RJA, IJA, and 24-month language.

## 4 | METHODS

### 4.1 | Participants

Participants came from the Infant Brain Imaging Study (IBIS), a prospective longitudinal study of infant siblings at high familial risk, and therefore high likelihood (HL), for ASD by virtue of an older sibling with ASD, as well as low likelihood (LL) infant siblings with an older sibling without ASD (Estes et al., 2015). The inclusion of children at high and low familial likelihood for ASD allows a broad range of developmental outcomes (Estes et al., 2015), including ASD in approximately 20% of HL children. Exclusion criteria are listed in the Supporting Information.

Data collection occurred at four study sites: University of North Carolina at Chapel Hill; Children’s Hospital of Philadelphia; Washington University in St. Louis; and University of Washington. Analyses included a mixed longitudinal sample of 741 infants (515 HL infants and 226 LL infants). Four hundred and twenty infants contributed three time points of data (at ages 6, 12–15, and 24 months), 218 infants contributed two time points of data, and 103 infants contributed only one time point. Trained clinicians provided a clinical best estimate diagnosis at 24 months for all infants based on assessment which included the Autism Diagnostic Observation Schedule (ADOS; Lord et al., 2000) and review of the DSM-IV-TR checklist (American Psychological Association, 2000). Ninety-six infants received an ASD diagnosis. The LORIS data management platform (Das et al., 2016) served as the study’s clinical and imaging hub for data collection, curation, preparation for analysis, and archiving. All parents provided written informed consent and protocols were approved by site Institutional Review Boards. Final sample demographics are shown in Table 1.

### 4.2 | Measures

#### 4.2.1 | Identification of potential indicators of social motivation at 6 and 12 months

The IBIS battery was reviewed for items querying social behavior in established parent-report instruments (those considered are described below) which index individual differences in infancy and represent parental impressions of behavior across time and contexts. Candidate social-motivation items, collected at 6 and 12 months, were selected based on face validity for Chevallier et al. (2012) definition of social motivation as the disposition to orient to social stimuli; seek, want, and like social contact; and work to maintain social interactions (Marrus et al., 2017; Marrus et al., in press). Items involved common, readily observable infant and toddler behaviors, including directing of attention to social stimuli, positive affect in an interpersonal context, initiation of social contact, or behaviors likely to sustain interaction.

As described in a separate manuscript from our group validating an initial series of items in a social motivation composite (Marrus et al., in press), item selection was initiated by the senior author, who selected a preliminary series of parent-report items as candidate indicators of social motivation from available 6- and 12-month measures. At 6 months, parent-report items were considered from the Vineland Adaptive Behavior Scales (VABS; Sparrow et al., 1984), a measure of adaptive function, and the Infant Behavior Questionnaire-Revised (IBQ-R; Gartstein & Rothbart, 2003), a measure of temperament. Example VABS items are “Shows affection to familiar persons (e.g., touches, hugs, kisses, cuddles, etc.)” and “Makes or tries to make social contact.” Example IBQ-R items include “How often during the last week did the baby enjoy watching while you, or another adult, playfully made faces?” and “When in a crowd of people, how often did the baby seem to enjoy him/herself?”

For the 12-month time point, in addition to the VABS and IBQ-R, parent-report items were considered from the Macarthur-Bates Communicative Development Inventory (MB-CDI; Fenson et al., 2006), a measure of expressive and receptive language and gesture use, and the First Year Inventory (FYI; Baranek et al., 2003), a screener for early risk markers of ASD and other neurodevelopmental conditions. Example items include, “Waves bye-bye on his/her own when someone leaves,” from the MB-CDI and, “Does your baby smile when looking at you?” and “Does your baby seem interested in other babies his or her age?” from the FYI.

Candidate items at this stage were next tested for internal reliability of these preliminary items using Cronbach’s alpha, and items lowering internal reliability at 6 or 12 months were eliminated. A second face validity check was performed by three co-authors (Marrus et al., in press) who reached a consensus, resulting in 22 6-month indicators and 40 12-month indicators. Items were again reviewed for face validity by the first author of the present study. As a preparatory step for MNLFA, a series of single-factor CFA models were conducted across groups divided by sex, likelihood, and age. Standardized loadings accommodated the different scales of the items. The final 23 items for this study were identified based on good model fit in the context of high loadings on a single social motivation factor, maximized overlap across 6- and 12-month items, and sufficient indicator-level variation (see Supplement for details).

#### 4.2.2 | Joint attention assessment

Initiating joint attention (IJA) was measured by coding the infant’s acts used to direct another’s attention based on the Communication and Symbolic Behavior Scales–Developmental Profile behavioral assessment (CSBS-DP; Wetherby et al., 2002), which is designed to measure social and communicative behaviors in infants and toddlers. The 30-min interaction is divided into six sampling opportunities: (1) wind-up toy, (2) balloon, (3) bubbles, (4) jar, (5) books, and (6) play. In question 7 of the CSBS, infants were scored based on the number of sampling opportunities in which they demonstrated at least one IJA bid, coded as an act used to direct another’s attention. IJA was based on scores obtained at either 12 or 15 months of age. Question 7 has been used as a measure of IJA in past publications (Eggebrecht et al., 2017).

Responding to joint attention (RJA) was measured using the Dimensional Joint Attention Assessment (DJAA; Elison et al., 2013; I. Stallworthy et al., 2021), a play-based assessment of infants’ abilities to respond to attention-sharing cues from adults. This assessment provides infants with four trials that each comprise up to four different attention-sharing cues that vary in cue redundancy, or sophistication. The least sophisticated, most redundant cue involves a gaze shift, head turn, point, and verbal cue while the most sophisticated, least redundant cue involves a gaze shift and head turn only (I. Stallworthy et al., 2021). Each infant receives a mean score averaged across all four trials that represents their RJA ability (Table S1). RJA was based on scores obtained at either 12 or 15 months of age.

#### 4.2.3 | Language measures

Twenty-four-month language outcomes were measured using the receptive and expressive subscale t-scores of the Mullen Scales of Early Learning (MSEL; Mullen, 1995). The MSEL has an Early Learning Composite as well as five subscale scores (visual reception, fine motor, gross motor, receptive language, and expressive language) and was collected at 24 months of age.

### 4.3 | Analytic strategy

#### 4.3.1 | Aim 1: Latent model of social motivation

##### Social-Motivation Indicators.

First, we fitted polychoric confirmatory factor analysis (CFA) models in R Studio using the *lavaan* package (Rosseel et al., 2021) to identify indicators of a single latent factor of social motivation. We first established configural invariance by conducting CFAs separately by group: by age (6 and 12 months), sex (males and females), and ASD likelihood (HL and LL). We eliminated items that did not exhibit sufficient variation for any of the groups and examined modification indices with values over 10. Modification indices to allow for covariance between items were scrutinized one at a time and accepted only within-measure, as it is reasonable that items drawn from the same assessment measure may share variation over and above that captured by the latent social-motivation construct. Given known developmental change in the first year of life and the flexibility afforded by MI computation, we allowed items to differ by age. Loadings and model fit were assessed using multiple commonly used indicators of relative (Tucker-Lewis index [TLI], Comparative Fit Index [CFI] with 0.9–0.95 considered good fit), and absolute (RMSEA with < 0.5 excellent fit, < 0.08 moderate fit; SRMR < 0.08 good fit) goodness-of-fit. Reasonable model fit for a single factor would be consistent with social motivation as a quantifiable superordinate construct based on shared variation from indicators representing different measurement orientations (e.g., adaptive functioning and temperament).

##### Measurement Invariance (MI).

Next, to establish MI for the indicators of latent social motivation derived from the previous step, we used moderated nonlinear factor analysis (MNLFA; Bauer, 2017; Bauer & Hussong, 2009; Gottfredson et al., 2019). Items in a latent model exhibit MI if their distribution depends only on values of the latent variable, and not on other characteristics of the individual (e.g., sex, age, ASD likelihood). Conversely, a given measure may be *non-*invariant, or exhibit measurement bias, if it functions differently for different groups of participants at a given level of the latent variable.

**FIGURE 1:**
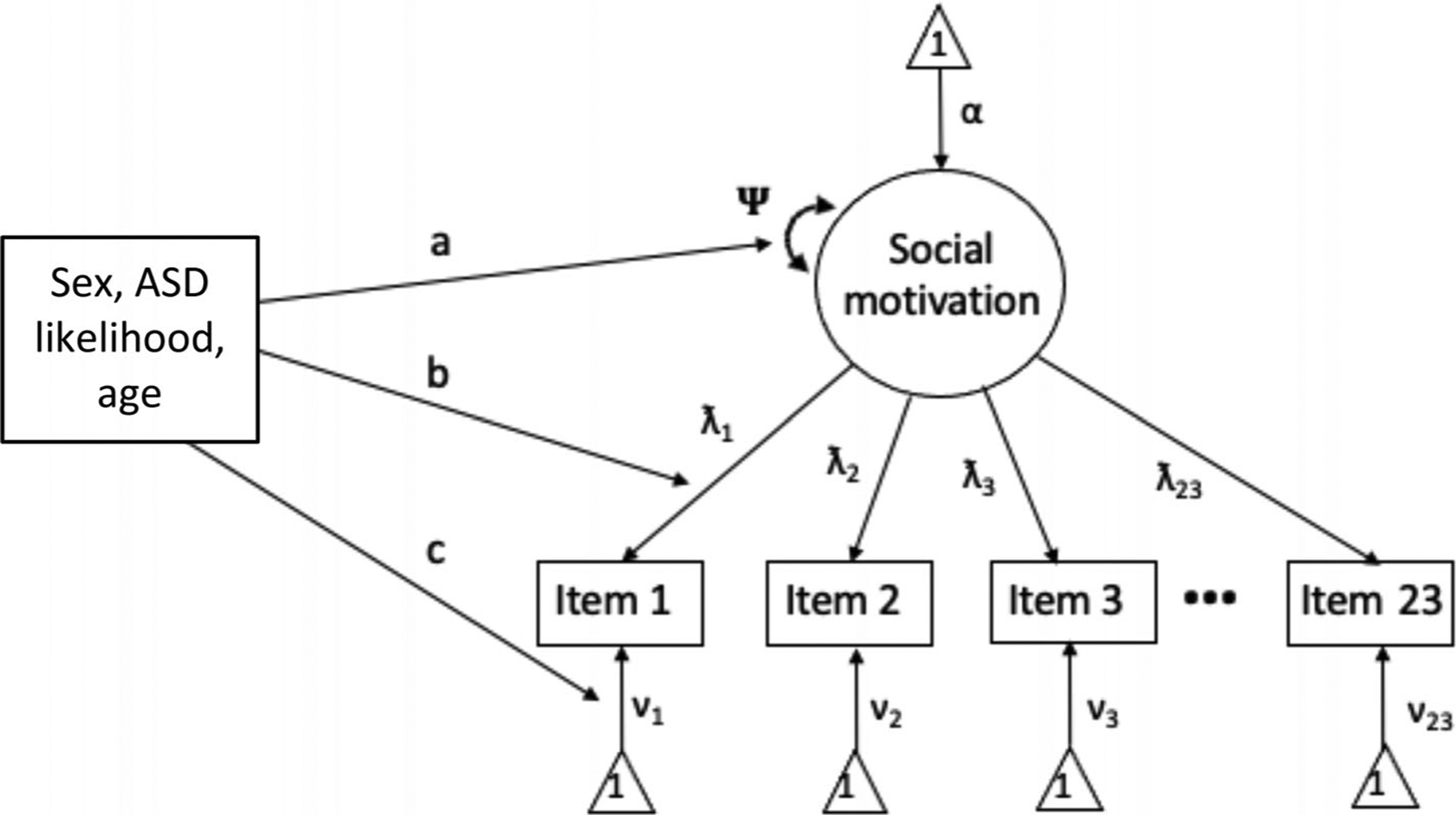
A latent social-motivation factor (circle) that gives rise to multiple observed indicators (squares) such as items from questionnaires. MNLFA assesses for measurement *non-*invariance (i.e., DIF) as a function a set of covariates (sex, ASD likelihood, age) that can manifest as associations *b* and/or c, which refer to loading and intercept DIF, respectively. Mean impact, which reflects associations between the covariate set and true levels of social motivation, shown as association *a*, can also exist following DIF correction. All covariates are assessed separately for DIF and mean impact

We used the *aMNLFA* (Gottfredson et al., 2019) and *MplusAutomation* (Hallquist & Wiley, 2018) packages in RStudio to assess and adjust for any measurement bias by sex, age, and ASD likelihood when creating social-motivation factor scores (Curran et al., 2014). More specifically, *aMNLFA* is a highly flexible, nonlinear latent factor model that tests and corrects for any differential item functioning (DIF) by a specified set of covariates (Bauer, 2017; Bauer & Hussong, 2009; Gottfredson et al., 2019). DIF refers to the case when two individuals of similar level do not endorse an item in the same way (Bauer, 2017), reflecting measurement *non*-invariance. It is assessed by evaluating the extent to which a set of (discrete or continuous) covariates moderate factor means and variances as well as item intercepts and factor loadings in the latent factor model (see Figure 1) (Curran et al., 2014). Items may exhibit nonlinear relations with item parameters and latent factors (Bauer & Hussong, 2009). Importantly, this approach allowed different item indicators of social motivation to be included for different ages, provided some item overlap was present across the age groups.

To assess and adjust for DIF, *aMNLFA* implements the following calculations using both RStudio and *MPlus* software (Muthén, L., & Muthén, B., 1998). First, given the longitudinal nature of the social-motivation items measured at 6 and 12 months, it draws a calibration sample of independent observations randomly sampled from the full dataset. Second, it assesses initial mean impact by regressing factor means and variances on the covariates (i.e., sex, age, likelihood). Next, it assesses initial DIF by testing covariate (i.e., sex, age, likelihood) effects on the factor loadings and intercepts, one item at a time as shown in Figure 1. Fourth, the algorithm tests all mean impact (Figure 1 association *a*) and DIF (Figure 1 associations *b* and *c*) effects simultaneously to form the final scoring model using the significant effects established in the previous steps and implements Benjamini-Hochberg correction for multiple comparisons to create the most parsimonious model. Finally, it obtains parameter estimates for all significant sources of DIF to create a final latent social-motivation scoring model that then generates social-motivation factor scores for each infant in the entire sample at 6 and 12 months of age. We assessed mean impact by familial likelihood, age, and sex; variance impact by age; and included HL, age, and sex as covariates (see Figure 1).

#### 4.3.2 | Aim 2: Developmental relations of social motivation in the first year

##### Social-Motivation Growth in the First Year.

Using the social-motivation factor scores at 6 and 12 months, we created a latent measure of change in social motivation from 6 to 12 months. Latent change score techniques are commonly used to represent change between two time points (Kievit et al., 2018; McArdle & Hamagami, 2001). This approach deconstructs and re-represents a single trajectory as latent change score such that the average change is reflected by the intercept of latent change variable. This allows us to evaluate the effects of both social motivation at 6 months and growth in social motivation from 6 to 12 months.

##### Examining Developmental Relations.

We then built upon the above model to simultaneously model the effects of 6-month social motivation and change in social motivation from 6 to 12 months, RJA and IJA at 12–15 months, and receptive and expressive language at 24 months. We used a structural equation model (SEM) framework, as it allowed us to simultaneously model multiple dependent variables, estimate latent variables, model residual covariance, and capitalize on full information maximum likelihood (FIML) to accommodate missing data. For instance, with this framework, we can account for known relations between joint attention and language in addition to the relations with social motivation. Standardized beta weights are based on the variances of both the observed and latent variables.

#### 4.3.3 | Missing data

Missing data were assumed to be missing at random (MAR)—that is, conditionally missing, given the other variables included in the observed covariance matrix (Enders, 2010). A strength of both the MNLFA and SEM approaches employed here is the ability to accommodate different patterns of missingness and use all available data using full information maximum likelihood (FIML) (Enders & Bandalos, 2001).

## 5 | RESULTS

### 5.1 | Aim 1: Establishing a latent model of social motivation

#### 5.1.1 | Social-motivation indicators

CFAs fitted to the different groups of age, sex, and familial likelihood status found that 23 items loaded sufficiently onto a single latent social-motivation factor for all groups (CFI = 0.91–0.98, TLI = 0.89–0.98, RMSEA = 0.01–0.06, SRMR = 0.08–0.14). Fit statistics by group are shown in Table 2 (see Figure S1 in the Supporting Information for loadings by group). These items spanned the different parent-report questionnaires, with 12 items from the IBQ-R, 6 from the VABS, 4 from the FYI, and 1 from the MCDI. Eleven items overlapped across ages, leaving 6 unique items each at both 6 and 12 months of age. All constituent elements of social motivation (liking, orienting, seeking, and maintaining) were represented (see Table 3, e.g.).

#### 5.1.2 | Measurement invariance

*aMNLFA* revealed significant DIF, or measurement *non-*invariance, for 11 of the social-motivation indicators spanning the different social-motivation aspects, as shown in Table 4. Nine items exhibited DIF by age, two by familial likelihood, and one by sex. Invoking “partial” measurement invariance (Byrne et al., 1989), these sources of DIF were adjusted for to create social-motivation factor scores. The final scoring model also indicated significant mean impact associations between the latent social-motivation variable and both familial likelihood (*B* = −0.274, *SE* = 0.096, *p* = 0.004) and age (*B* = 0.066, SE = 0.021, *p* = 0.002). This suggests that, on average, HL infants exhibited lower = − social-motivation scores compared to LL infants and social-motivation scores increased with age, after adjusting for all DIF. The final *aMNLFA* scoring model can be found in Figure S2 of the Supporting Information. One high familial likelihood observation was removed as an outlier (>4 SDs below mean). Factor scores were continuous and unimodal with a range of variation. The distributions of the final social-motivation factor scores that exhibit MI at 6 (M = −0.066, SD = 0.86) and 12 months (M = 0.38, SD = 0.81) are shown in Figure S3 of Supporting Information.

### 5.2 | Aim 2: Developmental relations of social motivation in the first year

**FIGURE 2:**
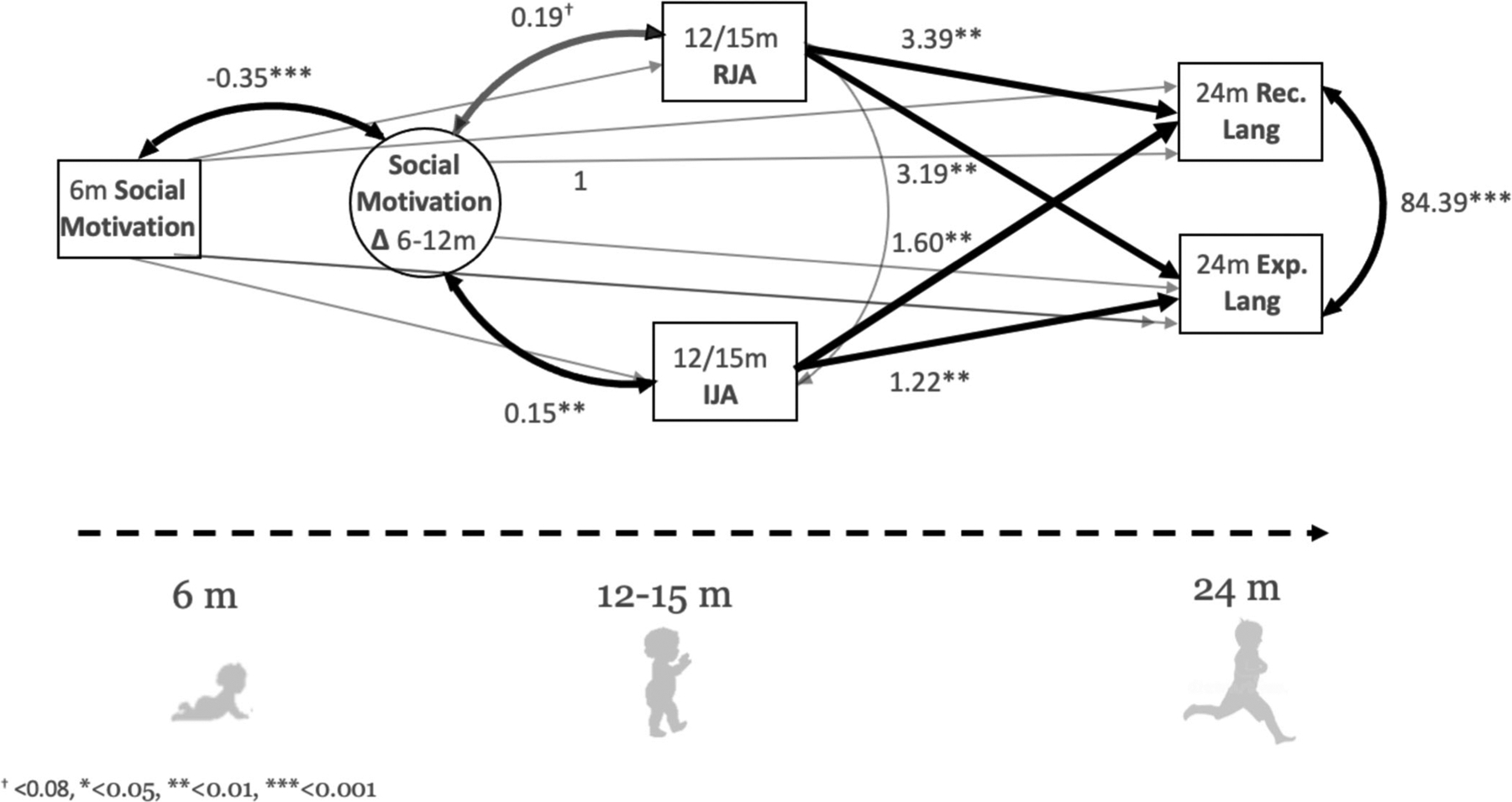
Significant associations are shown in bold arrows. 12-month social-motivation levels were used to create the latent change scores but are not pictured in for ease of interpretation

Six and 12-month social-motivation values were correlated at *r* = 0.61. The final SEM model simultaneously modeling relations between social motivation, RJA (M = 2.39, SD = 1.19), IJA (M = 1.62, SD = 1.55), receptive language (M = 49.88, SD = 13.98), and expressive language (M = 47.82, SD = 12.74) is shown in Figure 2 (−2 loglik = −12,691.68, AIC = 12745.68, BIC = 12869.05). Results show that, on average, infants increased in their social motivation from 6 to 12 months by 0.50 units (*p* < 0.001; *B*_*std*_ = 0.63; as indicated by model intercept values). A negative relationship between 6-month social motivation and 6–12 month growth in social motivation was observed, in which, on average, higher 6-month social motivation was associated with lower 6–12 month growth, and lower 6-month social motivation was associated with higher 6–12 month growth (*B*_*std*_ = −0.47, *p* < 0.001).

A significant relationship between 6- to 12-month growth in social motivation and IJA was observed. On average, infants who exhibited greater increases in social motivation from 6 to 12 months demonstrated significantly greater IJA by 0.15 units (*p* = 0.004; *B*_*std*_ = 0.12) and RJA by 0.19 units (*p* = 0.062; trend-only; *B*_*std*_ = 0.20) measured between 12–15 months, regardless of social-motivation levels at 6 months of age. We also found significant positive associations between IJA and both receptive (*p* = 0.001; *B*_*std*_ = 0.18) and expressive (*p* = 0.008; *B*_*std*_ = 0.15) language, as well as between RJA and both receptive (*p* = 0.001; *B*_*std*_ = 0.31) and expressive (*p* = 0.001; *B*_*std*_ = 0.32) language.

This model showed no statistically significant associations between social-motivation growth and either receptive or expressive language at 24 months of age (*p’s* > 0.55). Additionally, there were no statistically significant relations between 6 or 12-month social motivation and either joint attention or language (*p’s* > 0.45).

A secondary analysis, in which sex was added as a covariate, showed that all identified relationships remained significant, without substantive changes in magnitude (Supplement Figure S4). An exploratory analysis, shown in Figure S5 of the Supporting Information, revealed that in a model including only social motivation and language terms, 6- to 12-month change in social motivation was (positively) associated with future expressive (*p* = 0.011, *B*_*std*_ = 0.15) and receptive (*p* = 0.004, *B*_*std*_ = 0.16) language abilities when joint attention was omitted from the original model.

## 6 | DISCUSSION

Early social motivation has been hypothesized to provide a foundation for social communicative development, although this notion has been difficult to test given the challenge of establishing measures of social motivation in infancy. The advancements from the present study are twofold: (1) we demonstrate the feasibility of a latent model of social motivation in the first year of life, drawing on items from common parent-report measures, that exhibits measurement invariance for a heterogenous sample of infants; and (2) we provide some evidence for cascade effects for the development of social motivation in infancy and future joint attention abilities for HL and LL infants.

### 6.1 | Aim 1: Establishing a latent model of social motivation

This study extends past work identifying indicators of social motivation in infancy (Marrus et al., 2017; Marrus et al., in press) to confirm a theoretically driven latent model of social motivation representing multiple elements of social behavior underlying the disposition to engage with others (Chevallier et al., 2012). We found that items drawn from common parent-report questionnaires, spanning distinct elements of social motivation, loaded reasonably onto a single latent factor of social motivation. Further, we validated this latent model using a dimensional approach in a sample of infants of varying ASD familial likelihood, age, and sex by ensuring reasonable CFA fit and establishing MI.

Similar to other longitudinal work examining constructs early in development (e.g., Sifre et al., 2021), we found and corrected for measurement non-invariance in 11 out of 23 of our social-motivation indicators, signifying that at a given level of social motivation, these items behaved differently based on the age, sex, and familial ASD likelihood of participants, and that in future studies of social motivation, it is important to consider how social motivation may be expressed differently early in development as a function of these characteristics. For items exhibiting DIF, the most common source was age, suggesting that the function of several social-motivation indicators may change from 6 to 12 months as infants gain more skills and demonstrate their social motivation in more complex ways or in different contexts. For example, the indicator from the IBQ-R indexing enjoyment of closeness during feeding exhibited intercept DIF revealing that, on average, it is endorsed less frequently with age despite similar levels of social motivation, which may reflect infants progressing to the highchair for mealtimes as well as increasing ability to self-feed. At the same time, this item exhibited positive loading DIF with age. This indicates that infant enjoyment of closeness during feeding was a stronger indicator of latent social motivation in older versus younger infants, possibly also given older infants’ growing independence during mealtimes. In the case of familial ASD likelihood, intercept DIF was observed for the IBQ-R item rating the infant’s enjoyment of being held after the caregiver’s absence indicating that on average, for a given level of social motivation, parents of HL children endorsed this behavior less often than parents of LL children. In the case of sex, intercept DIF was found for a VABS item that indexes imitation of simple movements. This suggests that for a given level of social motivation, this form of imitation, which integrates social and motor capacities, is more likely to be endorsed in female than male infants, on average. This finding aligns with existing work suggesting that newborn females may exhibit greater fine motor movement and imitative gestures than males (Nagy et al., 2007). Adjusting for the DIF of these items prevented differences in age, sex, or likelihood from masquerading as variation in social motivation itself within our model investigating developmental relations.

### 6.2 | Aim 2: Developmental relations of social motivation in the first year

In partial support of our hypotheses, we found some evidence for cascade effects, or those spreading over time and domain, for early social motivation. Infants who exhibited greater intra-individual growth in social motivation between 6 and 12 months of age also exhibited significantly more IJA behaviors at the end of the first year of life, which in turn, were associated with greater 24-month language skills. Growth in social motivation was negatively associated with 6-month social motivation, illustrating that infants with low social motivation exhibited on average greater growth in social motivation than infants with high 6-month social motivation, consistent with an observed upper limit to the distribution of social motivation scores (Figure S3 in the Supplement) or potential regression to the mean.

While social motivation exhibited intra-individual growth, it also exhibited some trait-like inter-individual stability (i.e., rank-order stability) in infants, with *r* = 0.61, a level comparable to established traits including IQ, with *r* = 0.63 (Plomin & Deary, 2015) and personality, with *r* = 0.55 (Caspi et al., 2005). At this developmental stage, our findings demonstrated that intra-individual growth in infant social motivation, and not social motivation at 6 or 12 months, was associated with IJA. It is possible that more rapid growth in social motivation during this early developmental period may afford opportunities for social interactions in which children direct others’ attention as well as respond to caregivers’ attention towards them. High social motivation may contribute to infants’ use of pointing and other gestures to capture and direct the attention of others and augment the reward value of such interactions. Rapid growth in social motivation could help infants attune to others’ social cues, increasing opportunities for rewarding experiences involving shared attention. Such positive experiences could, in turn, both reinforce a child’s expression of social motivation and encourage caregivers to provide richer interaction contexts.

Of note, the potentially cascading effect of social-motivation growth takes place during a known sensitive period for social brain and behavior development (Maurer & Werker, 2014). The second half of the first year of life is marked by an explosion of social engagement (Tomasello, 2014), with increases in looking at the faces and social features of others (Frank et al., 2009, 2012), including sensitivity to the eyes in the context of gaze sharing (Brooks & Meltzoff, 2015); increases in gaze coordination (Northrup & Iverson, 2020) and anticipatory eye movements while observing action (Falck-Ytter et al., 2006); and increasingly complex visual exploration of social scenes (I. C. Stallworthy et al., 2020). The period from 6 to 12 months has also been found to be a particularly sensitive window for the experience-dependent narrowing of perceptual systems to attune to the most relevant social stimuli (e.g., Kuhl et al., 2006; Werker & Tees, 1984), as well as rapid brain development (see Shultz et al., 2018), including neural specialization for processing relevant social stimuli (Halit et al., 2003; E. Jones et al., 2015). Collectively, these findings suggest that social motivation from 6 to 12 months may interact with emerging social cognitive skill domains during a period when social behavior is highly sensitive to experience. This developmental context may help explain why 6- to 12-month growth in social motivation, rather than early 6-month social motivation levels, contributed to a cascade effect.

The importance of growth in social motivation, and its potential role in a developmental cascade in this period, is also consistent with the pre-diagnostic emergence of signs of ASD. Our findings suggest that while growth in social motivation may enhance developmental outcomes, decreases or plateauing of the level of social motivation during this period may contribute to a course of increasingly atypical development. Relative to ASD, W. Jones and Klin (2013) described increases (followed by plateauing) of attention to the eyes of others for typical infants but steady decreases from 2 to 24 months of age for infants who go on to develop ASD (W. Jones & Klin, 2013). Estes et al. (2015) reported trajectories of relative decreasing cognitive abilities for infants who developed ASD from 6 to 24 months compared to increasing patterns of scores for other groups (Estes et al., 2015). These findings parallel other work with HL and LL infants that reveals the importance of hyper expansion of brain cortical surface area from 6 to 12 months, which precedes the subsequent brain volume overgrowth associated with ASD-related social impairment (Hazlett et al., 2017). Our findings are thus aligned with a body of work supporting the importance of leveraging opportunities within this developmental period to support outcomes.

While growth in 6- to 12-month social motivation was associated with IJA, we did not confirm our hypothesis for RJA, which showed only a trend-level relation with social motivation. The finding for IJA supports existing theory (e.g., Tomasello et al., 2005) that in typical development earlier, more rudimentary social dispositions in infancy may promote experiences furthering development of future joint attention capacities. Differing relations between social motivation and IJA and RJA may reflect distinct trajectories of IJA and RJA during typical development (Mundy et al., 2007; Mundy & Jarrold, 2010). RJA and IJA may also entail partially overlapping aspects of social information processing (Mundy & Jarrold, 2010; Mundy & Newell, 2007), and similar to existing work (Bottema-Beutel, 2016), RJA was more strongly related to language outcomes than IJA. Additionally, within older typical populations, IJA has been found to uniquely recruit reward-based neurocircuitry (Nichols et al., 2005; Schilbach et al., 2010). This latter association (Mundy, 2018), along with findings supporting IJA’s more protracted typical development (Mundy et al., 2007) and more enduring clinical impairment in ASD (Dawson et al., 2004; Mundy et al., 1994) are congruent with the observed association of change in infant social motivation and IJA. We also note that our RJA and IJA measurement approaches differed, with RJA being assessed by sophistication of cues eliciting a response and IJA being assessed by frequency, and the trend-level RJA finding suggests the analysis may not have had sufficient power for small effects.

We also did not identify separate, direct associations of social motivation with receptive or expressive language in this developmental model. In an exploratory analysis, we could detect significant associations between 6-to-12-month growth in social motivation and 24-month language measures when joint attention was not included, suggesting that relations between social motivation and language were not significant over and above the relations between joint attention and language, consistent with the role of social motivation as part of a developmental cascade. Lastly, we observed no significant relations between social-motivation levels at 6 months and either joint attention or language abilities. This further suggests that in the case of 24-month language skills, growth in social motivation during the first year of life, with associated cascade effects on IJA, contributes to future language development. This finding underscores the importance of intensive longitudinal designs, rather than only cross-sectional designs, to monitor the course of early development. It also raises the possibility that early surveillance of social motivation could inform opportunities for interventions to stimulate socially rewarding experiences within the first year, which in turn could support the development of joint attention and improve functional communication outcomes. Future work will investigate the systems underlying social-motivation growth in the first year of life, including its neurophysiological, experiential, and genetic underpinnings.

## 7 | LIMITATIONS

Findings from this study should be interpreted in light of several limitations. Given that our social-motivation items were derived from a dataset with different items at each age, some indicators of social motivation differed by age or were unique to a specific age. While using all available indicators maximized detectable variation in social motivation during development, we could not fully disaggregate age-related versus indicator-related changes in social motivation and thus only established partial MI. In our mixed longitudinal design, some infants contributed data at a subset of time points or provided concurrent rather than serial data on social motivation and JA metrics at 12 months. These issues limited our interpretation of directionality of relations, although by maximizing subject inclusion, our approach increased statistical power and precision of model estimates within a more representative sample. Relatedly, our correlational study design does not allow causal claims about the relations between the constructs. Replication in more socio-demographically diverse study populations with dense longitudinal designs and developmentally continuous indicators of social motivation is warranted to clarify relations between social domains and infant developmental outcomes.

This study also relied only on parent report, a subjective measurement approach. While parent report is established in child clinical practice (e.g., for language; Fenson et al., 2006) and infant behavioral research (e.g., temperament; Gartstein & Rothbart, 2003), with some evidence for agreement with direct assessment (L. E. Miller, Perkins, et al., 2017), combining parent report with direct, objective methods is important for comprehensive measurement of social motivation that is less vulnerable to rater bias. Experimental paradigms testing social motivation could also offer greater precision for measurement of social motivation as well as evaluate contextual factors that might influence the demonstration of social motivation.

Lastly, item indicators in our measure could not differentiate the extent to which a behavior with face validity for social motivation reflects an infant’s disposition to engage socially or social skill, and real-world manifestations of social motivation could include both elements. Our leveraging a latent modeling approach allowed us to extract common variation related to social motivation across diverse indicators drawn from developmental questionnaires designed for different purposes. Future psychometric work on social motivation will benefit from incorporating diverse measurement modalities and approaches, including clinician-ascertained and biological indices. Incorporation of a greater array of social metrics than available in our existing dataset will also allow additional validity testing to investigate relative convergence and divergence with other aspects of social behavior, as well as measurement invariance for domains that support general function, such as cognition. Studies in larger atypically developing samples, including those with cognitive delays, will also allow follow-up evaluation of the generalizability of the measurement of social motivation and its developmental associations.

## 8 | CONCLUSIONS

In conclusion, this study offers a novel theoretically consistent approach to quantifying social motivation and explicating its role in early development. Findings provide support for a latent model of social motivation based on parent-reports of infants’ tendencies to orient socially and to seek, like, and maintain social engagement within a heterogeneous sample of infants. Developmental modeling adjusting for measurement non-invariance of several indicators by age, sex, and ASD likelihood reveals associations between social-motivation growth during the first year and future joint attention abilities for HL and LL infants. Together, these findings further our understanding of social motivation early in ontogeny with implications for future research investigating the neural bases of early social motivation, its role in ASD, and potential early screening and intervention targets.

## Supplementary Material

supplement

## Figures and Tables

**TABLE 1:** Final sample participant table

	HL	LL	TOTAL
*n*	509	225	734
Mean age in months (SD)	7.80 (2.44)	7.0 (1.43)	7.54 (2.22)
Income			
25–50k	106	40	146
50–100k	171	85	256
100k+	176	74	250
N/A	46	19	65
Ethnicity			
Hispanic	32	12	44
Race			
Asian	6	2	8
Black	15	14	29
White	420	178	598
Native Hawaiian/Pacific Islander	1	0	1
More than one race	55	25	80
N/A	2	3	5

**TABLE 2:** Fit statistics for the CFAs by group

Age	6				12			
Likelihood	HL	LR	LR&HR	M	HR	LR	LR&HR	
Sex	M&F		F		M&F		F	M
Chi square	160.84, *p* = 0.005	87.22, *p* = 0.16	120.16, *p* = 0.009	214.76, *p* < 0.01	202.52, *p* < 0.01	129.06, *p* = 0.023	149.85, *p* = 0.029	177.31, *p* < 0.001
CFI	0.97	0.97	0.98	0.91	0.97	0.98	0.98	0.96
TLI	0.97	0.97	0.97	0.89	0.96	0.98	0.98	0.96
RMSEA	0.04	0.03	0.05	0.06	0.06	0.04	0.048	0.06
SRMR	0.11	0.08	0.14	0.13	0.084	0.13	0.098	0.08

*Note*. CFAs were fitted by age, likelihood, and sex to establish configural invariance for a single latent factor reflected by the social-motivation indicators, a prerequisite for testing for MI using MNLFA.

**TABLE 3:** Example social-motivation item indicators

Questionnaire	Description	SM component
**VABS**	Returns a smile	Maintaining
**IBQ-R**	Enjoys closeness during feeding	Liking
**FYI**	Orients to people talking	Orienting
**VABS**	Makes/attempts social contact	Seeking

**TABLE 4:** Social-motivation items that exhibit DIF (corrected for via MNLFA)

Social-motivation aspect	Item description	Measure	DIF covariate	DIF type	Estimate (SE)
Liking	Enjoys closeness during feeding	IBQ-R	Age	Intercept	−0.117 (0.030)
			Age	Loading	0.078 (0.015)
Seeking	Snuggles after feeding	IBQ-R	Age	Intercept	−0.174 (0.035)
			Age	Loading	0.066 (0.024)
Liking	Enjoys watching adults make faces	IBQ-R	Age	Intercept	−0.062 (0.015)
Liking	Smiling during peekaboo	IBQ-R	Age	Loading	−0.058 (0.010)
Liking	Laughs during peekaboo	IBQ-R	Age	Intercept	0.068 (0.026)
			Age	Loading	−0.090 (0.017)
Maintaining	Imitates your sounds	IBQ-R	Age	Intercept	0.180 (0.022)
Liking	Enjoys being held after you’ve been away	IBQ-R	HL	Intercept	−0.223 (0.071)
Maintaining	Makes talking sounds when you talk to her	IBQ-R	Age	Loading	0.044 (0.015)
Liking	Smiles or laughs when you return		Age	Intercept	−0.054 (0.018)
		IBQ-R	Age	Loading	0.031 (0.011)
			HL	Loading	0.160 (0.076)
Liking	Enjoys herself in a crowd	IBQ-R	Age	Intercept	−0.057 (0.016)
Maintaining	Imitates simple movements (e.g., clapping)	VABS	Female	Intercept	1.251 (0.37)

## Data Availability

All data reported herein are available upon request.

## Highlights

- We describe a novel, theoretically based model of infant social motivation wherein multiple parent-reported indicators contribute to a unitary latent social-motivation factor.
- Analyses revealed social-motivation factor scores exhibited measurement invariance for a longitudinal sample of infants at high and low familial ASD likelihood.
- Social-motivation growth from ages 6–12 months is associated with better 12−15-month joint attention abilities, which in turn are associated with greater 24-month language skills.
- Findings inform timing and targets of potential interventions to support healthy social communication in the first year of life.
