# Supplementary material for: Quantifying latent social motivation and its associations with joint attention and language in infants at high and low likelihood for autism spectrum disorder
Source: Dev Sci. Author manuscript; Available in PMC 2023 Oct 23. (PMC10591497; doi:10.1111/desc.13336)
Supplement: supplement [file NIHMS1936365-supplement-supplement.pdf]

Supporting Information

Sample Exclusion Criteria

Exclusion criteria included: history of known genetic conditions or syndromes associated with ASD; significant medical conditions affecting growth, development, or cognition; sensory impairments such as significant vision or hearing loss; birth weight <2,000 grams and gestational age <36 weeks; history of significant perinatal adversity or exposure in utero to neurotoxins; contraindication for magnetic resonance imaging (MRI); predominant home language other than English; having a first- or second-degree relative with bipolar disorder, schizophrenia, or psychosis according to parent report; and having been adopted.

Item Selection

The 23 items used in the latent social motivation model were those that contained the most items overlapping at both age points as well as sufficient variation (i.e., it was statistically possible to fit the CFA) and loading in the series of single-factor CFA models fit to different groups of the data (Figure S1). We did not have collinearity issues with the CFAs but we did allow residual covariance for items that came from the same scale, as needed (e.g., two items from the IBQ-R). A correlation plot is shown below for the items that were collected concurrently. The final selection consisted of 12 items from the IBQ-R (11 at both 6 and 12 months), 6 items from the VABS (only at 6 months), 4 items from the FYI (only at 12 months), and 1 item from the MCDI (only at 12 months) for a total of 34 items across both age groups.

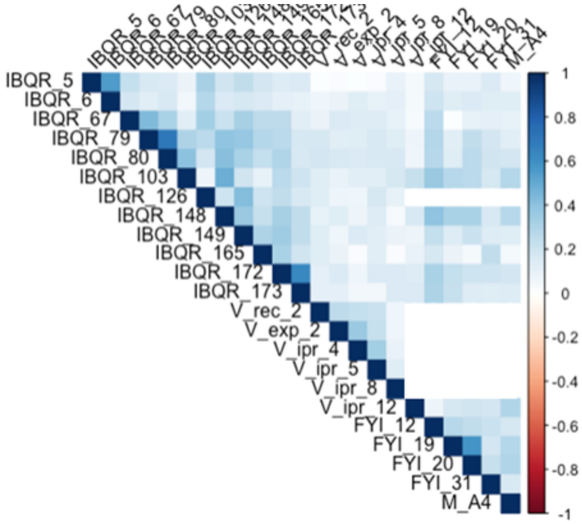

Potential Sources of Measurement *Non*-Invariance

Additionally, non-invariance as a function of sex could partially explain documented sex differences in early social behaviors (e.g., Chawarska et al., 2016; Maylott et al., 2021; Messinger et al., 2015; Nagy et al., 2007), and differences in the function of common early assessments for males and females (e.g., Estrin et al., 2020). There is some evidence that parents perceive social behaviors in their female infants differently from those of their male infants (e.g., Feldman & Reznick, 1996). Lastly, considering evidence of early social atypicalities in HL infants (e.g., Miller et al., 2019) and the possibility that parents with an older child with ASD may interpret and respond to questions about their infant's development differently than those without a child with ASD, measurement non-invariance could exist as a function of ASD likelihood status (HL vs LL).

Figure S1

CFA Loadings for all Social Motivation Items by Group

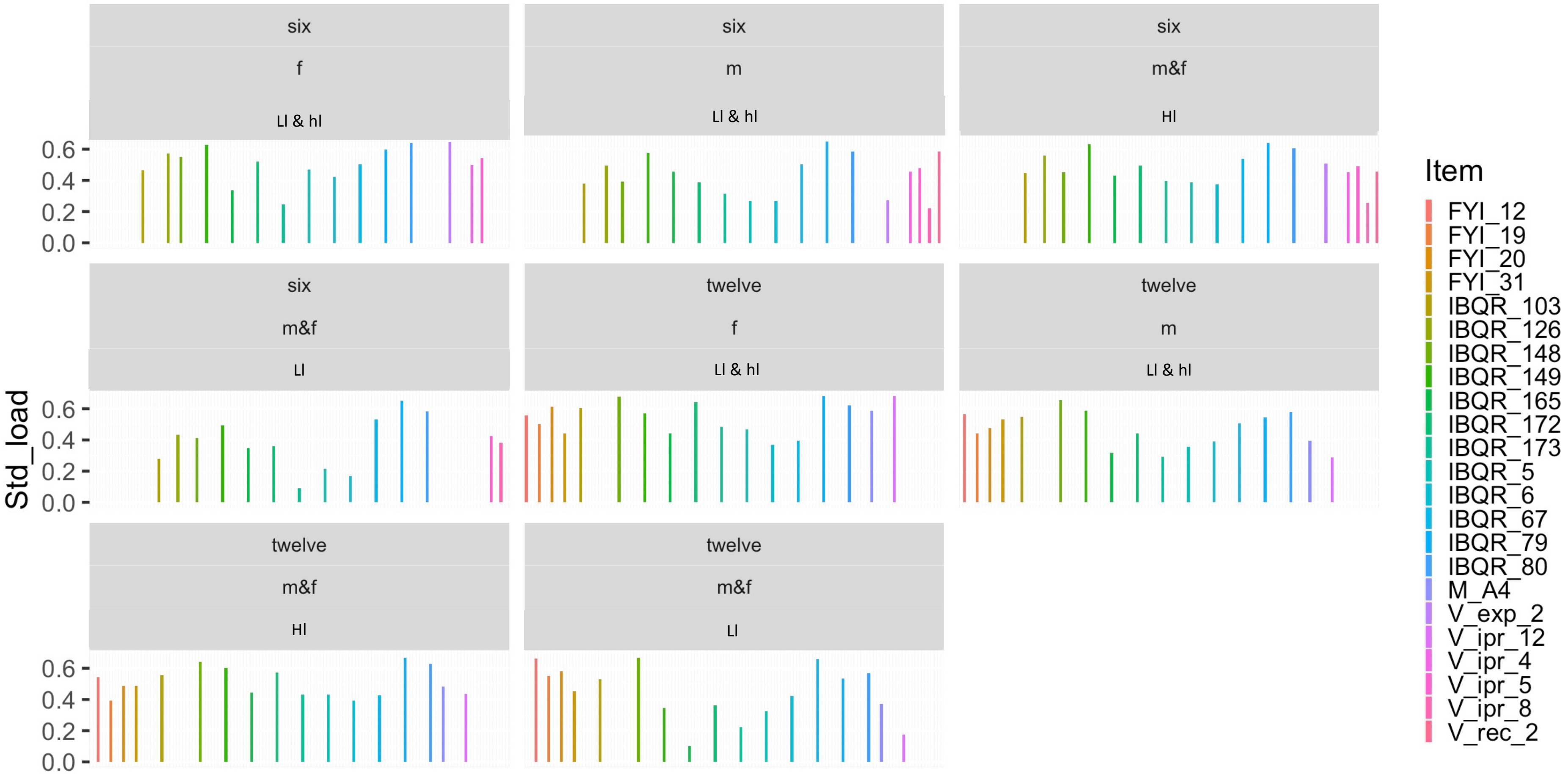

Note. Loadings are standardized. The top panel refers to the age group (six or twelve), the middle panel refers to the sex (male or female), and the bottom panel denotes familial likelihood (high or low). Colors refer to the different item indicators of social motivation.

Figure S2

MNLFA Final Scoring Model

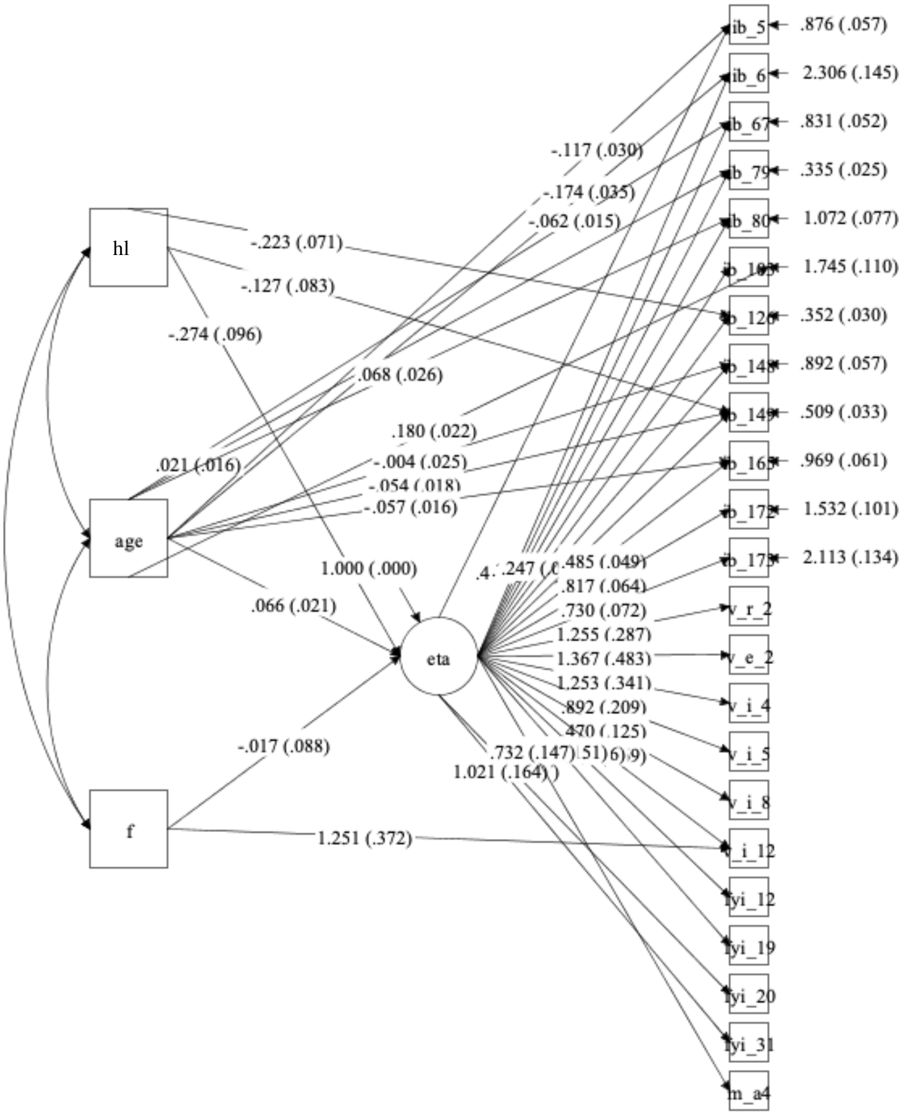

Figure S3

Distributions of Social Motivation Factor Scores at 6 and 12 Months

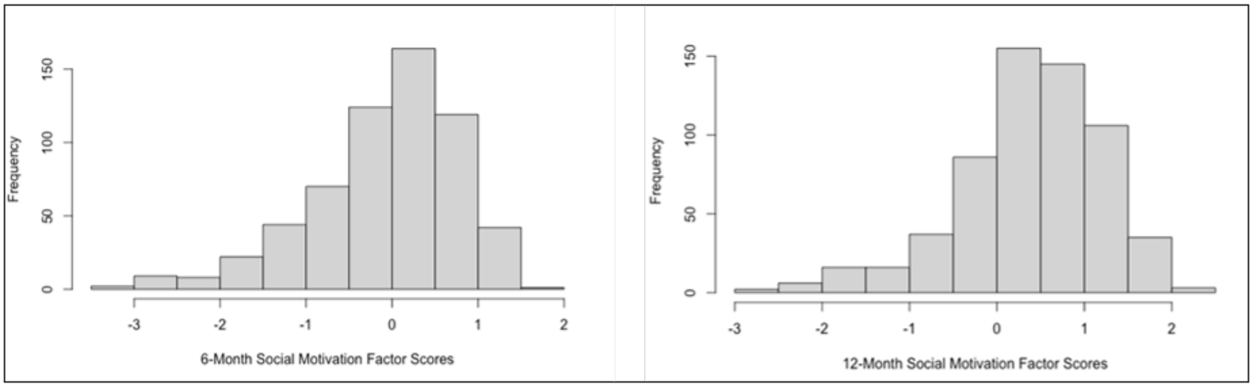

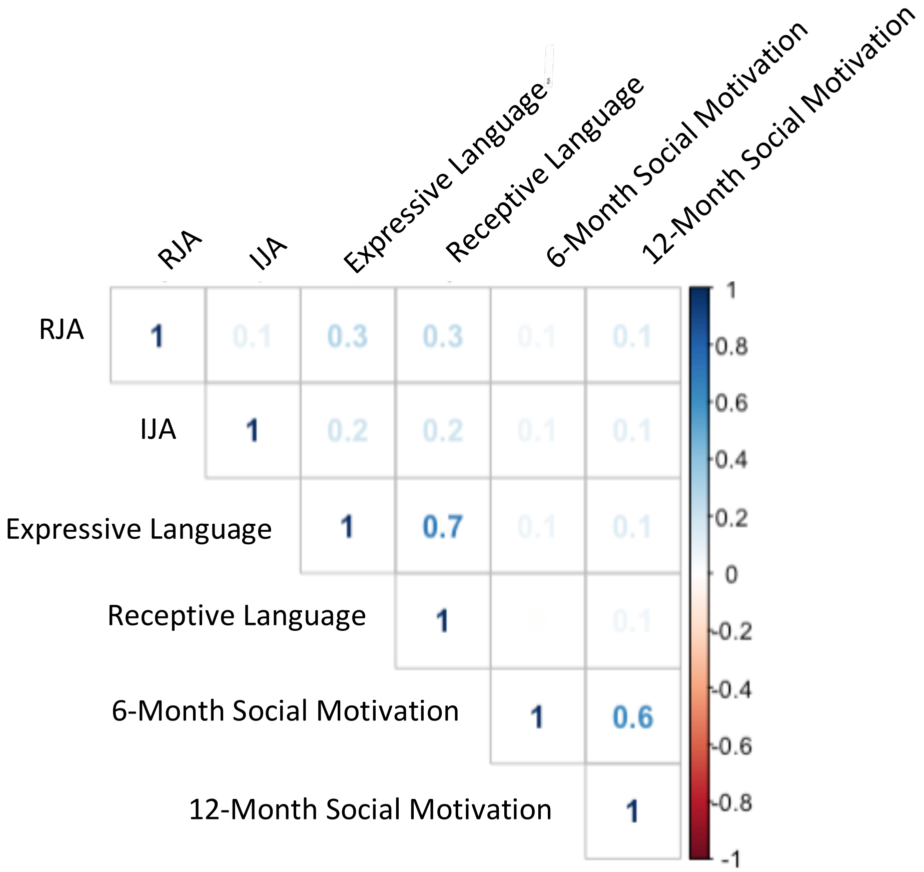

Figure S4

Path Model Secondary Analyses Including Sex as a Covariate

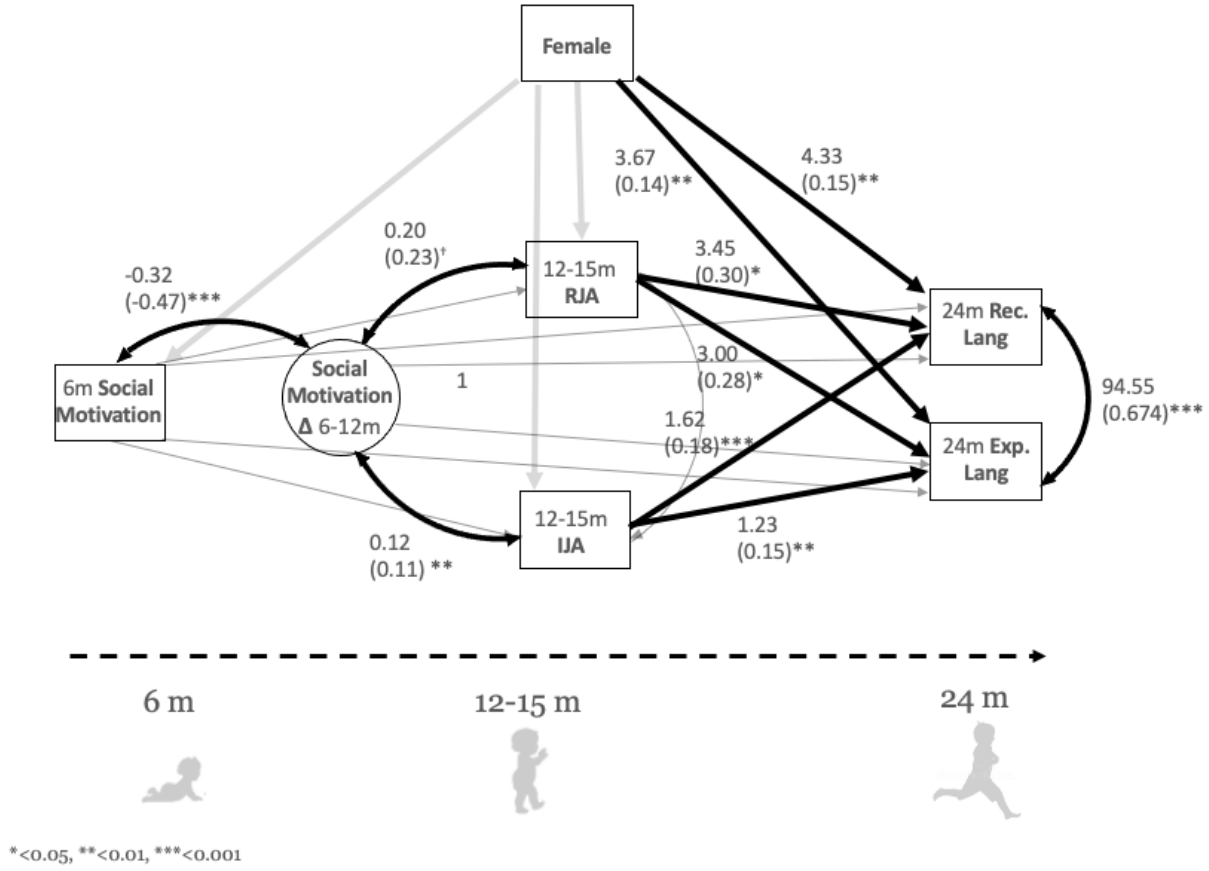

Note. Significant relations are bolded, and standardized values are provided in parentheses. 12-month social motivation levels and the effect of Female on 12-month levels (B=0.14, B_{std}=0.076*) are not pictured.

Figure S5

Path Model Exploratory Analyses Including Only Social Motivation & Language

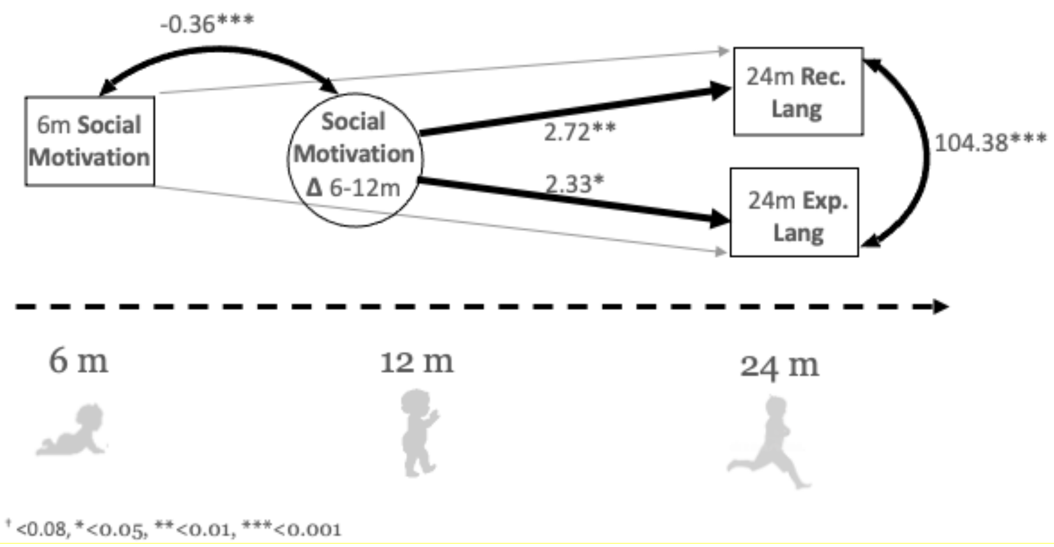

Table S1

Dimensional Joint Attention Assessment (DJAA) Scoring Guide

Bid number(s)	Cue redundancy level	RJA cue type	Infant response	Bid Score
1	Low	*Gaze shift* and *head turn* only	Looks in direction of target	4
2	Medium	*Gaze shift, head turn*, and *verbal cue*	Looks in direction of target	3
3	Medium	*Gaze shift*, *head turn*, and *point*	Looks in direction of target	2
4	High	*Gaze shift*, *head turn*, *point, and verbal cue*	Looks in direction of target	1
3-4	--	*Gaze shift*, *head turn*, and *point* OR *Gaze shift*, *head turn*, *point, and verbal cue*	Looks at the pointing hand of the experimenter	0.5
0-4	--	Any cue type	No response	0

Note. Table depicts the 4 different RJA cues of varying levels of redundancy (and the order in which they were delivered) given in each of the 4 trials, and the corresponding response resulting in each score (0-4). If in response to any of the cues the infant looks at the hand of the experimenter, a provisional score of .5 is given and the final press was attempted. If there is no response on the final press, .5 becomes the final score for that series. However, if the child responds in the direction of the object on the 4th bid, a score of 1 replaces the .5. A score of .5 could also be given on the final press if the infant looks to the hand of the experimenter. Trial scores were then averaged to create a mean DJAA score and a coefficient of variation (SD/M of trial scores).
